# Making sense of ultrahigh‐resolution movement data: A new algorithm for inferring sites of interest

**DOI:** 10.1002/ece3.4721

**Published:** 2018-12-26

**Authors:** Rhys Munden, Luca Börger, Rory P. Wilson, James Redcliffe, Anne Loison, Mathieu Garel, Jonathan R. Potts

**Affiliations:** ^1^ School of Mathematics and Statistics University of Sheffield Sheffield UK; ^2^ Department of Biosciences, College of Science Swansea University Swansea Wales UK; ^3^ Laboratoire d’Ecologie Alpine, UMR CNRS 5553 Université de Savoie Le Bourget‐du‐Lac France; ^4^ Office National de la Chasse et de la Faune Sauvage, Unité Ongulés Sauvages Gières France

**Keywords:** animal movement, biologging, high‐resolution data, movement ecology, site fidelity

## Abstract

Decomposing the life track of an animal into behavioral segments is a fundamental challenge for movement ecology. The proliferation of high‐resolution data, often collected many times per second, offers much opportunity for understanding animal movement. However, the sheer size of modern data sets means there is an increasing need for rapid, novel computational techniques to make sense of these data. Most existing methods were designed with smaller data sets in mind and can thus be prohibitively slow. Here, we introduce a method for segmenting high‐resolution movement trajectories into sites of interest and transitions between these sites. This builds on a previous algorithm of Benhamou and Riotte‐Lambert (2012). Adapting it for use with high‐resolution data. The data’s resolution removed the need to interpolate between successive locations, allowing us to increase the algorithm’s speed by approximately two orders of magnitude with essentially no drop in accuracy. Furthermore, we incorporate a color scheme for testing the level of confidence in the algorithm's inference (high = green, medium = amber, low = red). We demonstrate the speed and accuracy of our algorithm with application to both simulated and real data (Alpine cattle at 1 Hz resolution). On simulated data, our algorithm correctly identified the sites of interest for 99% of “high confidence” paths. For the cattle data, the algorithm identified the two known sites of interest: a watering hole and a milking station. It also identified several other sites which can be related to hypothesized environmental drivers (e.g., food). Our algorithm gives an efficient method for turning a long, high‐resolution movement path into a schematic representation of broadscale decisions, allowing a direct link to existing point‐to‐point analysis techniques such as optimal foraging theory. It is encoded into an R package called SitesInterest, so should serve as a valuable tool for making sense of these increasingly large data streams.

## 1 INTRODUCTION

The life track of an animal has the potential to reveal important information about its behavior, as well as the surrounding environment (Kays, Crofoot, Jetz, & Wikelski, 2015; Nathan et al., 2008). Modern, high‐resolution biologging data (≥1 Hz resolution) give insight into the fine‐grained structure of this life track (Bidder et al., 2015; Brown, Kays, Wikelski, Wilson, & Klimley, 2013; Noda, Kawabata, Arai, Mitamura, & Watanabe, 2014; Walker et al., 2015; Williams et al., 2017; Wilmers et al., 2015; Wilson, Shepard, & Liebsch, 2008). However, these data are often so big and detailed that extracting the important information is a formidable task.

Many studies have, in varying ways, suggested that the life track should be broken down into different scales, each representing different behavioral modes of animal movement (e.g., figure 1 in Nathan et al. (2008)). For example, state‐space modeling splits paths into predefined behavioral stages of movement, such as exploratory/encamped (Morales, Haydon, Frair, Holsinger, & Fryxell, 2004), foraging/migrating (Jonsen, Flemming, & Myers, 2005), or transient/resident (Patterson, Thomas, Wilcox, Ovaskainen, & Matthiopoulos, 2008). Behavioral changepoint analysis segments a path into sections with different statistical features (Buchin, Driemel, Kreveld, & Sacristán, 2011; Gurarie et al., 2016; Gurarie, Andrews, & Laidre, 2009) and can be used to classify these segments into distinct behaviors (Nams, 2014). Optimal foraging theory starts with the idea that paths can be described as movements either between or within foraging patches, and examines why animals make between‐patch movements at the particular times they have been observed to do so (Charnov, 1976; Pyke, 1984). There are also more general techniques for path segmentation that have arisen in subject areas beyond ecology (Demšar et al., 2015).

The modern era of high‐resolution data offers a great opportunity to make better inference of such behavioral modes. However, the sheer size of most modern data sets makes statistical analysis tricky to perform in a reasonable time frame. Furthermore, for a path where locations are recorded many times per second, the animal is often simply continuing to carry out a decision made some time previously. Therefore, an important part of the behavioral information is contained within a small subset of the data stream (Potts et al., 2018).

The development of techniques to infer behavioral decisions from high‐resolution data is thus timely and necessary. Here, we aim to describe an animal track as a sequence of “sites of interest,” which are areas where the animal spends a disproportionately long time, together with movements between these sites. Our algorithm breaks a long data stream down into a simple Markov‐process description of movement (similar to a “semantic trajectory” from movement analytics Demšar et al. (2015)), which has the potential to be analyzed using existing point‐to‐point techniques such as optimal foraging theory (Pyke, 1984) or step selection analysis (Avgar, Potts, Lewis, & Boyce, 2016; Fortin et al., 2005; Merkle, Fortin, & Morales, 2014). Our algorithm is based broadly on a site fidelity algorithm developed by Barraquand and Benhamou (2008) and Benhamou and Riotte‐Lambert (2012), but adapted for use with large, high‐resolution data. This adaptation requires finding ways of speeding up the algorithm, but we can take advantage of the fact that there is no need to interpolate between data points when they are only a few seconds apart, or less. We supply a method for assigning a level of confidence to our inference of the number of sites for an entire trajectory, displayed as a traffic‐light color. This indicates when further analysis may be necessary and gives an ad hoc goodness‐of‐fit test: something that is often missing from statistical studies of animal movement (Potts, Auger‐Méthé, Mokross, & Lewis, 2014).

We apply our algorithm to both simulated data, where the sites of interest are known, and dead‐reckoned 1 Hz tracks of cattle movement in the Alps (Bidder et al., 2015). For the latter data set, we already know two places that ought to be identified as sites of interest – a milking station and a watering hole. Thus, we can test both whether our algorithm can find these sites, and also if any other areas are uncovered that are of particular interest to the cattle. We show how our algorithm can be used to describe a complex movement path as a sequence of visits to sites and transitions between those sites. The algorithm is freely available as an R package SitesInterest, available as Supporting Information and also on CRAN (https://cran.r-project.org/web/packages/SitesInterest/index.html). This package will enable users to extract fundamental movement information from long, high‐resolution data streams.

## 2 METHODS

### 2.1 The “sites of interest” algorithm

Our algorithm uses a sliding‐disk method to infer areas of space where an animal spends most of its time (this is similar to the method used by Benhamou and Riotte‐Lambert (2012) to calculate “residence time”). In particular, our method is designed to be used on large sets (of order 10^5^ points) of ≥1 Hz resolution data. Like previous approaches (Barraquand & Benhamou, 2008; Benhamou & Riotte‐Lambert, 2012), our method involves sliding a disk of radius *R* along the animal’s path, looking for disks where the animal spends a disproportionately long time (see Figure 1 of Barraquand and Benhamou (2008) for a visual illustration). Modern high‐resolution paths can contain millions of locations. This is considerably more than those for which the algorithm of Benhamou and Riotte‐Lambert (2012) was developed (a few thousand). As such, this algorithm proves to be prohibitively slow for high‐resolution data (Supporting Information Appendix S1).

**Figure 1:**
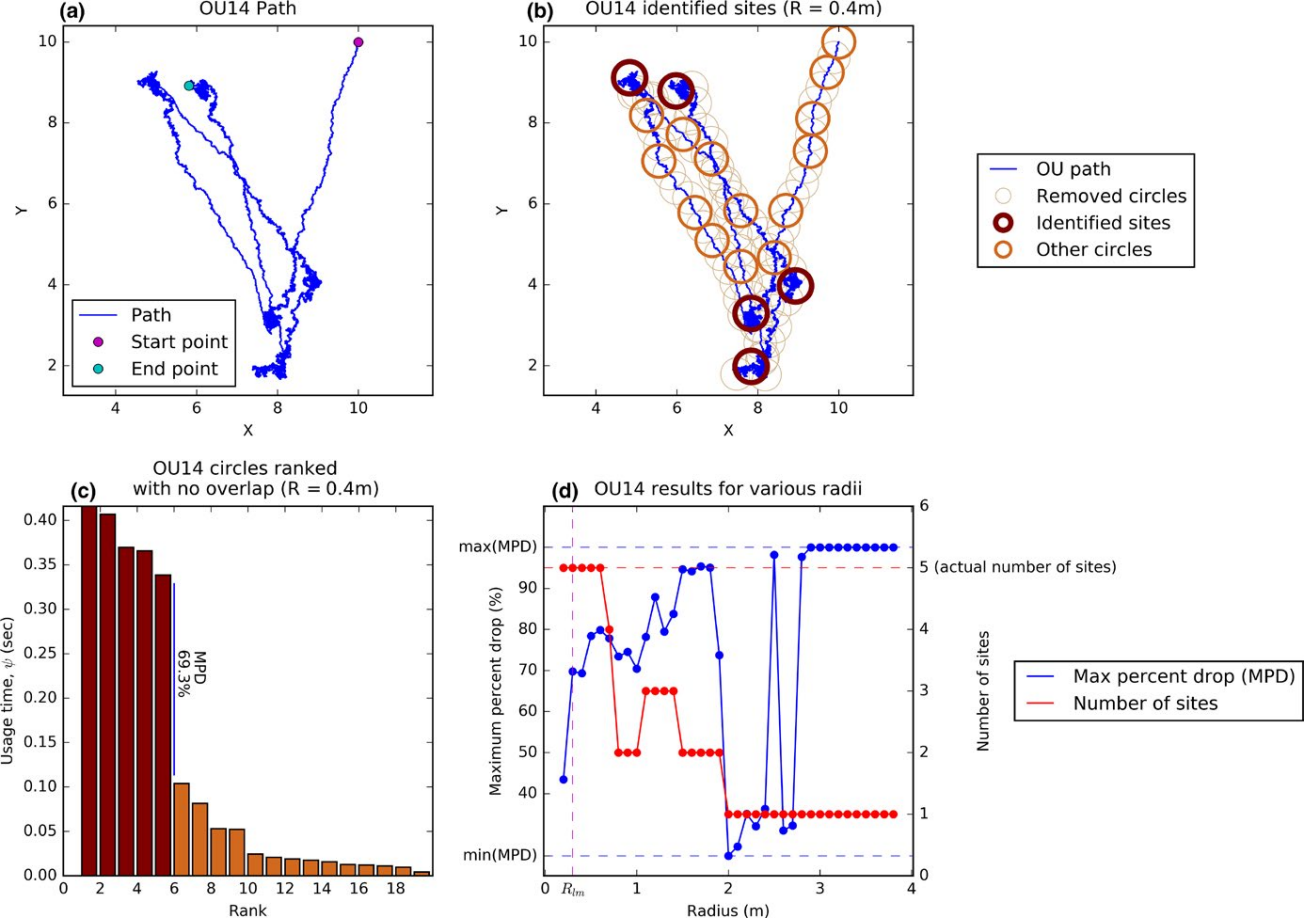
Demonstration of the algorithm applied to simulated data. Panel (a) shows the path of a switching Ornstein‐Uhlenbeck (OU) simulation (Simulation 14 in Supporting information Tables S3 and S7). Panel (b) shows the same path overlaid with the disks we examined for sites of interest. Maroon circles bound the disks identified as sites of interest. Of the remaining circles, those left after overlapping disks have been removed are given as orange colored and the others are yellow. Panel (c) gives a histogramme of the maroon and orange colored disks in ranked order. MPD is the value of the maximum percent drop. Panel (d) displays the maximum percent drop and number of identified sites as a function of the disk radius, *R*

To deal with this speed issue, we do two things, which we summarize here, leaving the details for Supporting information Appendix S1. First, we do not slide the disk over *every* recorded point in the path: potentially millions of disks. Rather, we start with a disk centered at the first data point, then each subsequent disk is centered at the first recorded location after the animal first leaves the previous disk, meaning that we only need to analyze a relatively small number of disks (approximately the length of the track divided by *R* for relatively straight trajectories and less if the tortuousity is higher). This dramatically reduces the number of disks examined by the algorithm, while ensuring all of the space that the animal covers is analyzed.

Second, when looking for the places at which the animal being studied entered and left a disk, we subsample our data at every *s*‐th location (see Supporting information Appendix S1). Once an entry‐ or exit‐point is identified, say between the *i*‐th and (*i* + *s*)‐th location, we use the full path between points *i* and *i* + *s* to identify the exact position of entry or exit. The larger we choose *s*, the quicker the algorithm. However, if we choose *s* to be too high, we are in danger of missing information if the animal moves in and out of a disk within *s* time steps. Therefore, there is a trade‐off in choice of *s*, which ultimately depends on the data being analyzed. For our 1 Hz data, we found that *s* = 10 gave rapid yet accurate results (Supporting information Tables S4 and S5).

Having calculated the *usage time* for each disk, defined to be the amount of time spent in each disk across the whole time‐period over which the path is measured, we rarefy the set of disks further by removing any disk that overlaps with another disk of higher usage time (Supporting information Appendix S1, Figure 1b). The salient information from the resulting collection of nonoverlapping disks is displayed in a histogramme of decreasing usage times (Figure 1c). This is superficially similar to a scree plot from principle component analysis, and we use similar ideas to analyse the plot (Jolliffe, 1986).

In essence, we want to find a point at which the heights of the bars in the histogramme “drop‐off” rapidly, separating out comparatively well‐used sites (to the left) from transitory ones (to the right). We look at each adjacent pair of bars on the histogramme for the greatest percentage difference in the usage times. This is referred to as the *maximum percent drop* (MPD). The sites of interest are defined to be disks corresponding to the bars to the left of this MPD (Figure 1c).

The resulting set of identified sites depends very much on the choice of *R*, the disk radius. As such, we need criteria to determine which value of *R* is “best” for accurate identification of sites. In practice, we found that no single criterion works perfectly in every situation. Instead, we give a technique for determining a value of *R*, together with a traffic‐light color (Red, Amber, Green) denoting the level of confidence we have in our algorithm having found the actual sites of interest for the animal, where Green is high, Amber is intermediate, and Red is low. We then suggest that the user supplements this with biological intuition, especially in the Red and Amber cases, to check that the algorithm has returned a reasonable estimate of the actual sites of interest.

The starting point for finding *R* is to calculate the MPD for a variety of different *R*s, plotted in Figure 1d, and look for the first local maximum of this graph, which we denote *R*_LM_. Local maximality suggests that the sites of interest can be identified more clearly with *R* = *R*_LM_ than with close‐by values of *R*. We choose the *first local* maximum, rather than the *global* maximum, because the MPD tends to 100% as *R* becomes large enough so that the most oft‐used disk contains almost all of the path. We then apply two further criteria.

The first criterion insists that the MPD must be greater than a predefined *threshold* value, *T*_MPD_. This can be chosen either as a fixed value or as T_MPD_ = min(MPD) + *k*(max(MPD) – min(MPD)), where *k* is a constant, referred to as the *adaptive threshold* value. Here, min(MPD) and max(MPD) are, respectively, the minimum and maximum MPDs for all values of *R* tested (see e.g., Figure 1d). Brownian motion simulations can be used to derive a lower bound for the threshold value (Supporting information Appendix S1).

**Figure 2:**
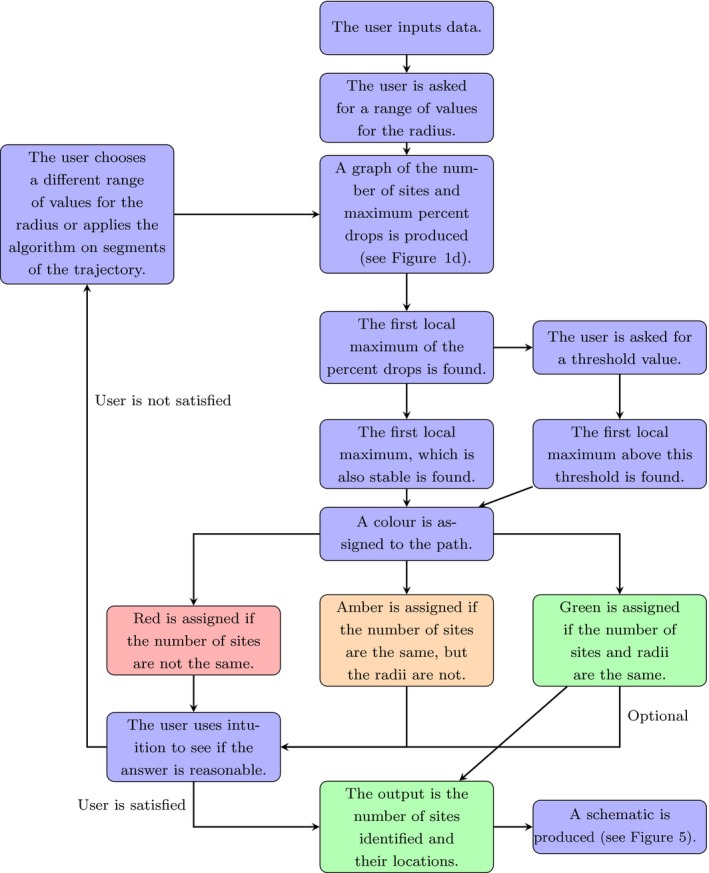
A flowchart describing how the algorithm is implemented

The second is a stability criterion, meaning that if the radius is changed slightly from R = R_LM_, the number of sites identified will remain unchanged. Based on the results of these two criteria, a color is assigned depending on the consistency between the results of using each criterion. The Green label is assigned if both criteria identify the same number of sites and radius value, Amber is assigned if they result in the same number of sites, but different radii and Red is assigned if the number of sites are different (see Supporting information Appendix S1 for more details). This gives a qualitative level of confidence in the algorithm's performance and could be used as a warning signal to suggest when further analysis would be helpful. The complete method for finding sites of interest is summarized in Figure 2.

### 2.2 Data

#### 2.2.1 Simulated data

To test the efficacy of our algorithm, we constructed a collection of simulated paths using a switching Ornstein‐Uhlenbeck (OU) process (Blackwell, 1997; Taylor & Karlin, 2014). At any point in time, an object following a switching OU process has a center of attraction toward which it is moving. However, there is also a certain amount of (Gaussian) randomness in the movement process (see Blackwell (1997) and Blackwell, Niu, Lambert, and LaPoint (2016) for more details on the switching OU process and applications to animal movement). In these simulations, the “real” sites of interest are defined to be the centers of attraction of the switching OU process.

**Figure 3:**
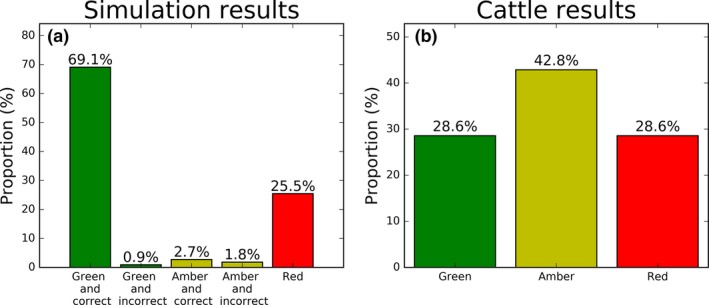
The proportion of paths assigned to each of the color categories for the switching OU simulations (Panel a) and daily cattle paths (Panel b). The numbers denote the percentage of sites assigned to each category

We ran 110 OU simulations in a box of 10 by 10 units, varying the number of points of attraction between 1 and 10. We also varied the positions of these points and the long‐term standard deviation about these points of attraction (i.e., the standard deviation of the stationary distribution of the OU process). Details are given in Supporting information Tables S6–S9, and 12 examples are shown in Supporting information Figure S6. We tested whether the algorithm correctly picks out these points of attraction as sites of interest (i.e., both that the number of sites is identified correctly and that these sites contain the centers of attraction of the switching OU process; Figure 3).

We ran each of the OU simulations through the algorithm with radii values ranging from 0.2 to 3.8 units with 0.1 units between consecutive values. The minimum radius value was chosen so that it was greater than the greatest distance between any two consecutive locations. The maximum radius value was chosen so that it would be larger than any potential site. Other than these constraints, the radii were chosen blindly so as to simulate having no prior knowledge about the trajectories.

#### 2.2.2 Cattle data

Cattle data were collected in July 2017 from a group of cows from the French Alps in the Bauges Mountains (Massif des Bauges, 45.61°N, 6.19°E). The cattle were tagged with Daily Diary tags (with triaxial accelerometers and magnetometers; Wildbytes Technologies http://www.wildbyte-technologies.com and Gipsy‐5 tags; TechnoSmArt Tracking Systems http://www.technosmart.eu), placed inside custom‐built 3D printed ABS plastic housings and attached to commercial nylon cow collars (Fearing Lifestyles, Durham, UK). The accelerometer readings were recorded at a frequency of 20 Hz approximately and 6 Hz for the magnetometer readings. Both were subsampled to 1 Hz, whereas GPS readings were recorded every 15 min. The path was then reconstructed using Framework4 (Walker et al., 2015), which uses the Dead Reckoning procedure (Bidder et al., 2015).

We focused on seven ten‐hour long paths. We ran each path through our algorithm with radii values ranging from 10 to 100 m, with 1 m between consecutive values. We suggest that the minimum radius used be at least half the body length of the animal, to have any biological meaning, and typically several times more than this. We also ran our algorithm over the entire collection of seven paths. The latter gives us information about sites that the cattle might return to day‐by‐day, whereas the former might reveal sites that are of interest to particular cows on specific days.

## 3 RESULTS

### 3.1 Simulated data

Our algorithm correctly identified sites of interest for 72% of our 110 simulated paths (Figure 3a). 69.1% of these paths were both correctly identified and given a Green level of confidence. The algorithm only misidentified one path with a Green output, so 98.7% of the 77 paths classified Green identified the correct number of sites. This suggests that if a Green output is given, we can be reasonably confident that the sites of interest have been identified correctly.

Of those assigned Amber, only two (1.8%) were falsely identified. For some of the simulations assigned to the Red category, using either the threshold criterion or the stability criterion returned the correct answer (see Supporting information Tables S10–S13). The results presented used a fixed threshold value of *T*_MPD_ = 65% as this minimized the number of incorrect Green paths.

### 3.2 Results from Cattle data

Figure 3b summarizes the results of running our algorithm over each of the seven cattle trajectories independently (see Supporting information Table S14 for the full results). These results came from using a fixed threshold value of *T*_MPD_ = 50%, which was chosen so as to minimize the number of paths assigned to the red category and was also greater than the lower bound found from the Brownian motion simulations (Supporting information Appendix S1). The running time for each trajectory (of 30–40,000 points) was less than a minute (Supporting information Table S1), whereas for all seven together (247,000 data points), it took just over 4 min and the algorithm appears to scale linearly (Supporting information Figure S1).

**Figure 4:**
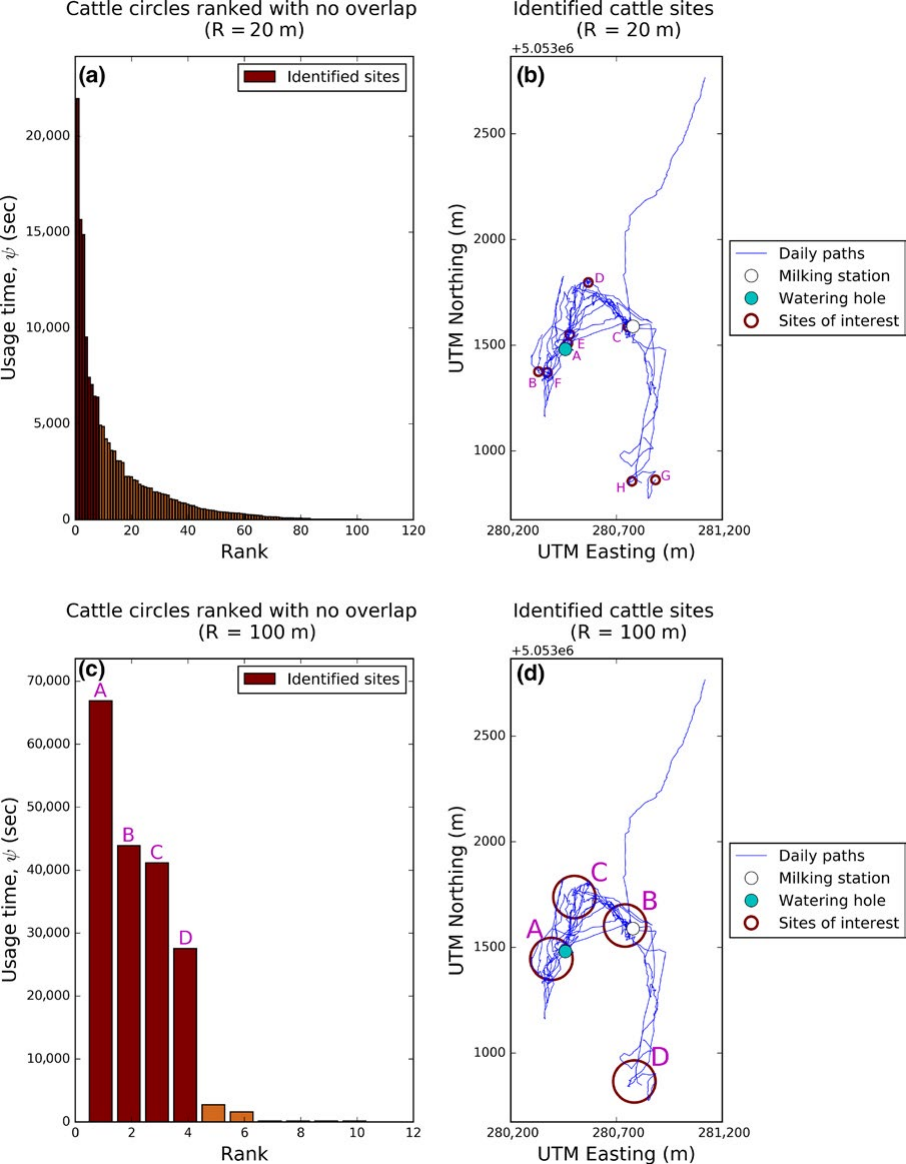
Identification of sites for seven paths of cattle movements obtained using a radius of *R *= 20 in Panels (a,b) and *R* = 100 in Panels (c,d). Sites of interest were identified from the bar charts, by sight for *R* = 20 and *R* = 100. The bars are labeled alphabetically, with A being the circle with the greatest usage time, all of which correspond to the maroon circles in the right hand plots

Although only two of the paths gave a Green level of confidence, running the algorithm over a single trajectory encompassing all seven paths reveals clear sites of interest (Figure 4). If we choose *R* = 20, a relatively fine‐grained value, there are substantial drops after the 1st and 3rd circles, but both of these missed out interesting information, such as the cattle's movements to the southeast. So instead we look at the drop between the 8th and 9th circles (Figure 4a,b, Supporting information Figure S7). In actual fact, the maximum percent drop occurs after the 83rd circle. However, the resulting set of circles is large and hence rather uninformative, so we define the sites of interest to be the first eight disks. Two of these (A and E) occur about the watering hole and one (C) about the milking station. We would expect cattle to use these two locations quite frequently (pieces of salt licks are provided for cows close to the milking station), so it makes sense that our algorithm identifies them as sites of interest. Importantly, our algorithm also reveals five other sites of interest in less‐expected places. This opens up the question of why the cattle are interested in these locations, and helps guide future data analysis to examining specific areas that seem to be valuable to the animals (e.g., habitat features and food availability).

If we use *R* = 100, a coarser‐grained value, we found six sites which again covered the majority of the path, so was not a very informative set of sites. However, from the histogramme, there is a substantial drop after four disks (Figure 4c). These encompass six of the eight sites identified by using *R* = 20, including both the milking station and the watering hole. It also suggests that the pair of sites (G,H) from Figure 4b might actually be a single site, and this warrants further field investigation. A similar lesson holds for the pair (A,E).

Although the *R* = 100 case is in some ways better than *R* = 20 since it recognizes the watering hole as a single site rather than two, its coarseness leads to a potentially missed site of interest in the middle‐left of the area (Figure 4b,d). The *R* = 20 case picks this out (sites B and F from Figure 4b). This suggests that visually examining the algorithm output for more than one value of *R* can be valuable.

**Figure 5:**
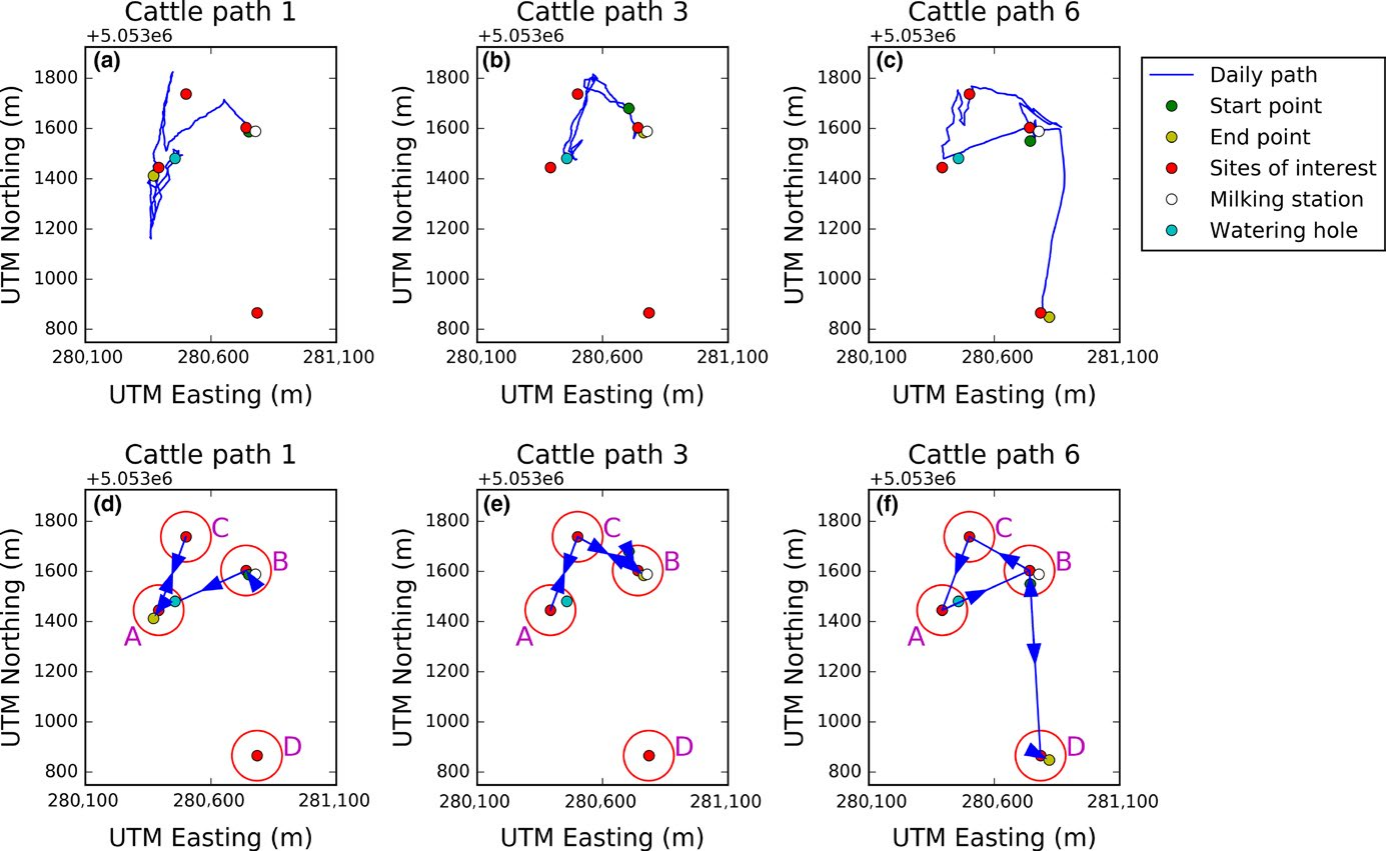
Three particular examples of cattle paths (a–c) with the corresponding schematic plots below (d–f). The schematics represent simplifications of the full path that highlight the broadscale movement decisions made by each cow. The centers of sites of interest are defined by the red dots and their boundary by the red hoops. The flowchart represents the movements of Cattle Path 6 between sites of interest. The letters represent the sites of interest, corresponding to the same letters in Panel (f). The number in brackets give the number of minutes the cow spends at that site for that particular visit. The arrows represent the cow moving from one site to the next, with the associated numbers representing the number of minutes the cow spends moving between these sites

As well as identifying sites of interest, our results enable simplification of a complex movement path into a schematic diagram reflecting the main behavioral decisions made by the animal. In Figure 5, we illustrate this with three example paths of cattle movement (see Supporting information Figure S8 for all seven). The sites of interest are those four identified in Figure 4d for the *R* = 100 case. This schematic breaks up a complex movement trajectory into a simple Markov process, enabling users to ask questions about why the animal transitions between the different sites at the times it does, which could be answered by using existing point‐to‐point techniques such as optimal foraging theory or step selection analysis. This is similar in flavor to the *semantic trajectories* defined by Yan, Chakraborty, Parent, Spaccapietra, and Aberer (2013).

Note that we can define the process of choosing patches such that the probability of an animal to either change or not change sites is based purely on the *current* state of both the animal and the environment. As such it can always be framed as a Markov process, whereby the decision to move at time *t* is based on the state of the system at time *t*. For example in Figure 5, suppose the cow is currently grazing at site C, but, at some point in time, becomes sufficiently thirsty to necessitate a move to the watering hole at Site A. Although the causal chain leading to this decision may be arbitrarily long, the decision to move from C to A is simply based on the present state of the animal (particularly thirst, but also maybe hunger, mobility etc.) and the environment (e.g., distance from C to A, effort or risk of moving from C to A and so forth).

## 4 DISCUSSION

This paper introduces an efficient algorithm for decomposing a long, high‐resolution data stream of animal locations into a simple Markov‐process description of animal movement decisions. We have applied our algorithm to both simulated and real data (Figure 3), showing that it is effective in recognizing known sites of interest, but can also reveal other, less‐expected places that the animal is visiting frequently (Figure 4). Such information opens up questions as to why each of these sites is particularly interesting to the animal, and why it makes the decision to move between these sites at the particular times it does. These latter questions can then be examined by existing point‐to‐point techniques, such as step selection analysis (Avgar et al., 2016; Fortin et al., 2005), conditional entropy (Riotte‐Lambert, Benhamou, & Chamaillé‐Jammes, 2016), sequence analysis methods (De Groeve et al., 2016), or optimal foraging theory (Pyke, 1984).

Unlike model‐based approaches, our algorithm makes no assumptions about why sites may be of particular interest, just that they are small areas which are well used in comparison with other areas of equal size. It is broadly based on previous works of Barraquand and Benhamou (2008); Benhamou and Riotte‐Lambert (2012) that find areas of high‐intensity usage by sliding a circle of fixed radius, *R*, along the path (similar questions were also addressed by Sila‐Nowicka et al. (2016)). However, the size and resolution of our data require that these algorithms be significantly adapted, which is a key contribution of our work, having increased the algorithm's speed by approximately two orders of magnitude. Movement ecology is increasingly dealing with such high (subsecond) resolution data, so such adaptations are becoming ever more valuable.

As well as applicability to higher‐resolution data, our algorithm has some qualitative differences to that of Benhamou and Riotte‐Lambert (2012) that are worth highlighting. These result from slightly different aims. Here, our interest is in finding patches that are used for a disproportionately large amount of time compared to other areas of the landscape. In contrast, Benhamou and Riotte‐Lambert (2012) seek to describe space use patterns more generally. As such, their work focuses on constructing various “heat maps” representing different aspects of space use, namely the Utilization distributions, Intensity distribution, and Recursion distribution (see Benhamou and Riotte‐Lambert (2012) for definitions of these quantities). For our aims, we found it more beneficial simply to identify high usage sites. That said, it may be beneficial in certain circumstances to perform some postprocessing of the identified sites to see if any are better‐described by noncircular geometries, for example, by using least cost paths (Long, 2016) to see if there are particular regions within a site which are less well‐used than others.

One of the challenges of developing such a window‐sliding algorithm is to determine the “correct” size of the window, *R*. Fauchald and Tveraa (2003) suggested using the log‐variance of the resident times between circles, to give a variance‐scale curve as a function of *R*. The maximum of this curve gives an indication of the ideal window size to use. This was met with several criticisms by Barraquand and Benhamou (2008). Nonetheless, Kapota, Dolev, and Saltz (2017) revisited the variance‐scale curve method and improved on it in several ways, specifically addressing the concerns of Barraquand and Benhamou (2008). In principle, these techniques could be used in combination with our usage‐time algorithm if the user is particularly concerned in identifying sizes of the sites of interest. However, we found that a combination of biological intuition and examining places where there was a clear drop in the usage time histogramme (Figure 1c) was a simple and effective method of doing the same job to a reasonable degree of accuracy.

Many of the existing statistical and theoretical tools available to movement ecologists were made when coarser data were the norm. As such, it is not always trivial to adapt these techniques to the new world of high‐resolution data. For example, many methods in the literature are based on distributions of step lengths and turning angles between successive data points (Avgar et al., 2016; Ironside et al., 2017; Morales et al., 2004). However, when the “steps” are only a fraction of a second apart, there are not a lot of sensible biological inferences that can be made about step‐wise “decisions,” as animals are unlikely to be making discrete decisions at such a high frequency. One other improvement has been the addition of the quantification of uncertainty (traffic‐light color assignment), which warns users when performing further checks would be appropriate. This is a novel aspect of our method that as far as we know, has not been used before. If the assignment comes up as “red” or “amber,” it may be valuable to investigate whether carefully chosen subsections of the path may give better inference. For example, if there is an overwhelmingly dominant site of interest (e.g., a sleeping site), it may be valuable to run our algorithm over periods of time when the animal is not likely to be asleep.

Once sites of interest have been identified, together with the transition points between them (Figure 5), a wealth of opportunity opens up for answering questions concerning routine movement behavior (Ironside et al., 2017; Peron, Fleming, Paula, & Calabrese, 2016). For example, Riotte‐Lambert, Benhamou, and Chamaillé‐Jammes (2013) examined periodicity within an animal's movement pattern and identified using wavelet analysis. The same authors later used conditional entropy to quantify the predictability of repeating movement patterns between sites of interest (Riotte‐Lambert et al., 2016). Questions related to trap‐lining, path recursion, and predator prey studies were reviewed by Berger‐Tal and Bar‐David (2015). All of these are forms of movement recursion that could make use of the sort of schematic descriptions of movement typified in Figure 5, especially if the paths are longer so the movement sequences contain more detailed information.

The algorithm's output also enable users to examine differences in the between‐ and within‐site movement patterns. These path segments can then be analyzed in isolation, for example, by identifying smaller‐scale turning points (Potts et al., 2018). In summary, our algorithm turns long, complicated streams of data into simple schematic decisions of broadscale behavioral decisions. This technique gives a foundational basis for tractable analysis of high‐resolution movement data.

## AUTHOR CONTRIBUTIONS

LB, RPW, and JRP conceived and designed the research; RM performed the research; LB, RPW, JR, AL, and MG provided data; RM and JRP led the writing of the manuscript. All authors contributed critically to the drafts and gave final approval for publication.

## DATA ACCESSIBILITY

Data used in this manuscript will be archived on FigShare with https://doi.org/10.6084/m9.figshare.7125614. Access to the data has been embargoed until 01/01/2020.
