# Supplementary material for: Making sense of ultrahigh‐resolution movement data: A new algorithm for inferring sites of interest
Source: Ecol Evol. 2018 Dec 26;9(1):265–74. doi: 10.1002/ece3.4721 (PMC6342090; doi:10.1002/ece3.4721)
Supplement: Supplementary file 1 [file ECE3-9-265-s001.pdf]

SUPPLEMENTARY MATERIAL FOR "MAKING SENSE OF VERY HIGH RESOLUTION MOVEMENT DATA: A NEW ALGORITHM FOR INFERRING SITES OF INTEREST"

Rhys Munden[1,a], Luca Börger[2], Rory P. Wilson[2], James Redcliffe[2], Anne Loison[3], Mathieu Garel[4] and Jonathan R. Potts[1]

1 School of Mathematics and Statistics, University of Sheffield, Hicks Building, Hounsfield Road, Sheffield, UK, S3 7RH.
a Email: rdmunden1@sheffield.ac.uk (Corresponding author)
2 Department of Biosciences, College of Science, Swansea University, Singleton Park, Swansea, Wales SA2 8PP, UK
3 Laboratoire d'Ecologie Alpine, UMR CNRS 5553, Université de Savoie, Bâtiment Belledonne, F-73376 Le Bourget-du-Lac, France
4 Office National de la Chasse et de la Faune Sauvage, Unité Ongulés Sauvages, 5 allée de Bethléem, Z.I. Mayencin, 38610 Gières, France

S1. Algorithm Comparisons

S1.1. **Runtime.** Here we compare the runtimes from using the "sliding circle" part of the algorithm of Benhamou and Riotte-Lambert (2012) (referred to as the "original algorithm") and our algorithm (referred to as the "adapted algorithm"). Both algorithms use a circle which slides along the path and the residence/usage time is calculated for each circle position. The residence and usage time are measures of the time spent in the vicinity of the circle and the absolute time spent in the circle, respectively. Also the original algorithm centres a circle at every point along the path, whereas the adapted algorithm does not. Despite missing some points, the adapted algorithm still ensures that the entire trajectory is covered. The original algorithm also checks for when the circle boundary has been crossed between all consecutive points, whereas the adapted algorithm checks over a fixed interval (of s steps) and then identifies the exact crossing time within this interval.

The average runtime over the seven cattle paths when using the original algorithm was 11 hours 10 minutes, where the minimum was 7 hours 11 minutes and the maximum was 13 hours 51 minutes. This was decreased when using the adapted algorithm for $s = 1$ to 5 minutes on average and was further reduced when $s = 10$ to an average of 32 seconds (Table S1).

The choice of s will change the runtime roughly at the same scale as the choice of s (i.e. if $s = 1$ it will be roughly ten times slower than if $s = 10$ as can be seen in Table S5). A larger s will also result in less accuracy, where the visits of duration less than s time steps could be missed (see Section S1.2).

Date: November 23, 2018.

	Path 1	Path 2	Path 3	Path 4	Path 5	Path 6	Path 7	Average
No. points	36913	39601	35964	36898	30605	31504	35736	35317.3
Original algo-rithm	11:22:31	12:56:32	13:51:53	11:08:42	08:29:20	07:11:02	13:20:59	11:11:34
Average ($s = 1$)	00:03:51	00:04:58	00:04:11	00:06:07	00:05:23	00:05:52	00:04:44	00:05:00
Average ($s = 10$)	00:00:39	00:00:31	00:00:26	00:00:39	00:00:34	00:00:37	00:00:18	00:00:32

Table S1. The runtimes (hour:min:sec) from applying the algorithm to cattle data for seven paths, using the original algorithm and the averages (over various radii) from using our algorithm with $s = 10$ (Table S4) and $s = 1$ (Table S5).

It should also be noted that the runtime will vary depending on how long the path is (Figure S1). The choice of radius will effect the runtime inversely, so a larger radius will take less time as less circles will be required to cover the path. The shape of the path, typically characterised by the tortuosity, will also have an effect, as this could mean that less circles are required to cover the path. As such we have applied our algorithm to a set of straight line trajectories, since a straight line maximises the number of circles required to cover the path. In order to maximise the number of circles this means minimising the amount of the path inside each circle. The path must also pass through the centre of the circle, meaning that the minimum path must be a line along the circle's diameter. The simplest way of arranging these circles is in a straight line. Therefore a straight line can be considered to be a worst case scenario.

We used a set of straight line trajectories to investigate the relationship between the runtimes of the two algorithms as the number of points increases. The total length of the path and radius were kept constant, but the number of points was varied. Figure S1 shows that the runtime of the adapted algorithm increases approximately linearly with the number of points, whereas for the original algorithm the runtime increases approximately quadratically. This is because more discs are needed for the original algorithm, whereas for the adapted algorithm the number of discs remains constant.

S1.2. **Accuracy.** As well as comparing the speeds of the two algorithms, we also compared their accuracy. Firstly we checked how changing the size of the interval (denoted by s) affected the accuracy, where this interval is used to check for circle entrances and exits.

Over the seven cattle paths we compared the accuracy of using $s = 10$ against $s = 1$ for ten radius values (10, 20, ..., 100m). Using $s = 1$ gives the usage time correct up to the resolution of the data, since this means that entrances and exits are checked for between all consecutive points. In 55 out of 70 cases, the position of the circles did not alter. A circle's position may change if the first exit from the prvious circle is for a short period of time and is therefore missed, resulting in the centre of the next circle being different. Over these 55 cases the average usage

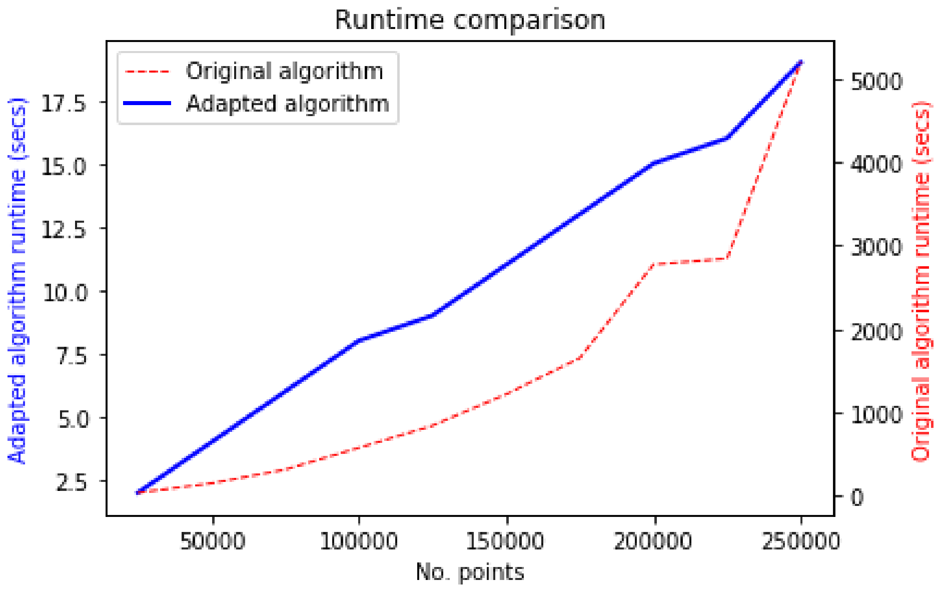

FIGURE S1. A comparison between the runtimes of the original and adapted algorithms for paths with different numbers of points, where the original algorithm is the algorithm of Benhamou and Riotte-Lambert (2012) and the adapted algorithm is our own one. It should also be noted that there is a significant difference in scale between the two y-axes.

time discrepancy was 0.26 seconds, which is less than the time taken for a single step (1 second). Also in 17 of these cases, we found that changing s made no difference to the usage time.

Due to the difference in output between our algorithm and the whole of the algorithm from Benhamou and Riotte-Lambert (2012) it is difficult to compare the accuracy of the two methods. The original algorithm gives a utilisation distribution, whereas our adapted algorithm gives clearly delimited areas. It also does not make much sense to use movement kernel density estimation on such fine-scale data, since we know exactly where the animal was almost all of the time. So we first subsampled the cattle data before applying the whole of the algorithm from Benhamou and Riotte-Lambert (2012).

As can be seen from Figure S2 there is only one site identified no matter which subsample rate is chosen. This method also misses the site to the South-East (site D in Figure 4.d) found from our algorithm and combines our other three distinct sites (sites A,B and C in Figure 4.d) into one.

It is also interesting to note that in the calculation of the Utilisation Distribution, there is no need to calculate the residence time. This means that the method of Benhamou and Riotte-Lambert (2012) is not sensitive to the choice of radius, like our method is. However, our algorithm identifies particular sites, so needs the radius to be able to define a boundary for these sites. Also we include the traffic-light scheme to help choose which radius to use.

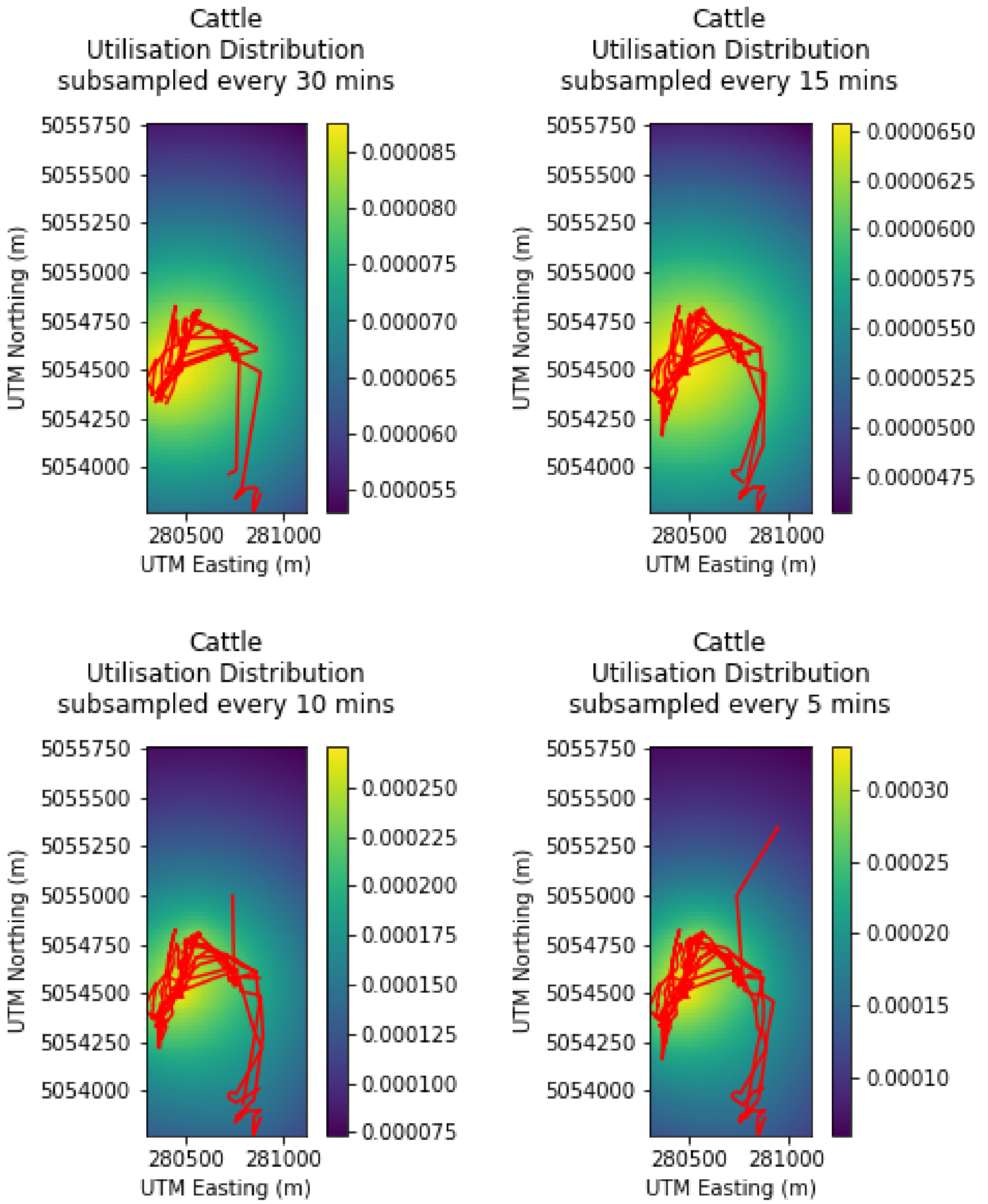

Figure S2. The Utilisation Distribution calculated using the algorithm of Benhamou and Riotte-Lambert (2012) on the cattle data subsampled and interpolated at different rates. These were calculated using Movement Kernel Density Estimation, where the smoothing parameters came from using the theory of Brownian Random Bridges.

S2. Details of the algorithm

The algorithm is split into several parts. The first part of the algorithm calculates the usage time for different areas along the path. Then from these data, a number of sites and other useful information is put out. This is then repeated for various radius values. From these, the 'best'

radius is chosen and the number of identified sites and their positions is the final output along with a colour representing the consistency in the output from using two different criteria.

S2.1. **Usage time.** The algorithm centres circles at particular points along the path of the animal, $(X(t^{(c)}), Y(t^{(c)}))$. The algorithm will calculate the usage time $(\psi(t^{(c)}))$, which is the time spent inside these circles. This usage time includes any time that the animal spends inside the circle on a second or third etc. visit, as such this is done by finding the times when the animal enters and then leaves the circle for each visit.

Passages through these circles are defined as either being forward passages or backward passages. We define forward passages through the circle to be ones occurring after the time point that the circle is centred on $(t^{(c)})$ and then backward passages are ones occurring before this time point. Each passage is defined by an entrance and exit time, which are the time points that the animal enters and leaves the circle.

Here we define the conditions necessary for the different crossings. Firstly we define

$$d\big((x_1, y_1), (x_2, y_2)\big) = \sqrt{(x_1 - x_2)^2 + (y_1 - y_2)^2}$$

This is the Euclidean metric and is our measure of distance between two points $\big((x_1, y_1)$ and $(x_2, y_2)\big)$.

Let $(X_k^{(c)}, Y_k^{(c)})$ be the centre of the circle we are looking at and s be a positive integer. Then a forward entrance occurs between t_i and t_{i+s} if

$$d\big((X_i, Y_i), (X_k^{(c)}, Y_k^{(c)})\big) \geq R \text{ and}$$
$$d\big((X_{i+s}, Y_{i+s}), (X_k^{(c)}, Y_k^{(c)})\big) \leq R$$

where $i \in \{k, k+s, k+2s, \dots\}$. The algorithm looks for the exact crossing time and this will occur between t_j and t_{j+1} if

$$d\big((X_j, Y_j), (X_k^{(c)}, Y_k^{(c)})\big) \geq R \text{ and}$$
$$d\big((X_{j+1}, Y_{j+1}), (X_k^{(c)}, Y_k^{(c)})\big) \leq R$$

where $j \in \{i, i+1, \dots, i+s-1\}$. The algorithm then chooses the crossing time to be the one out of these two points $((X_j, Y_j)$ and $(X_{j+1}, Y_{j+1}))$, which is closest to the circle's edge, meaning that

$$t^* = \begin{cases} t_j & \text{if } d\big((X_j, Y_j), (X_k^{(c)}, Y_k^{(c)})\big) \leq d\big((X_{j+1}, Y_{j+1}), (X_k^{(c)}, Y_k^{(c)})\big) \\ t_{j+1} & \text{if } d\big((X_j, Y_j), (X_k^{(c)}, Y_k^{(c)})\big) > d\big((X_{j+1}, Y_{j+1}), (X_k^{(c)}, Y_k^{(c)})\big) \end{cases}$$

where t^* is the identified time point of crossing.

For forward exits this same approach is repeated, except the inequalities are switched around. For backward entrances and exits everything is the same as for forward entrances and exits, except $i \in \{k, k-s, k-2s, \dots\}$ and $j \in \{i-s+1, i-s+2, \dots, i\}$.

Symbol	Meaning
$(X(t), Y(t))$	The position of the animal at time, t
(X_i, Y_i)	The position of the animal at time, t_i
t_i	Time points
T	Set of time points ($T = \{t_1, t_2, \dots t_N\}$)
s	The interval length the algorithm looks over
$(X(t^{(c)}), Y(t^{(c)}))$	The position of the centre of the circle at time $t^{(c)}$. ($X_j^{(c)} = X(t_j^{(c)})$ and $Y_j^{(c)} = Y(t_j^{(c)})$)
$t_j^{(c)}$	The time points at which the circles are centred at
R	The radius of the circles
R_{min}, R_{max}	Maximum and minimum of the range of values for R
r	Distance between consecutive R values
$\psi(t^{(c)})$	Usage time of circle centred at $t^{(c)}$
$F(t^{(c)})$ and $B(t^{(c)})$	The forward and backward passage time crossings for the circle centred at $t^{(c)}$
$\zeta(t^{(c)}), \phi(t^{(c)})$ and $\beta(t^{(c)})$	The total number of visits, number of forward visits and backward visits to the circle centred at $t^{(c)}$
MPD_R	The maximum percent drop associated with radius, R
$L(MPD_R)$	The first local maximum MPD
T_{MPD}	Threshold for the maximum percentage drop
R_0, R_1, R_2	The R values identified by using the first local maximum, a threshold value and the stability criterion respectively
$\hat{n}$	Actual number of sites
n_R	Number of sites identified from using a radius value of R

TABLE S2. Glossary of notation

The algorithm looks for crossings within the ranges $\{1, \dots, k \pmod{s}\}$ and $\{N - s + k \pmod{s} - N \pmod{s}, \dots, N\}$, where $k \pmod{s}$ is the remainder when k is divided by s and similarly for $N \pmod{s}$. This is necessary since without checking over these ranges the method will not find a crossing if it occurs less than s time steps from either end of the path. The algorithm also checks whether the path either starts or ends in a circle and this will be taken as a backward entrance or forward exit respectively.

S2.1.1. *Calculating the usage time.* For each circle the forward and backward crossing times will be stored in vectors

$$F(t^{(c)}) = [F_1(t^{(c)}), F_2(t^{(c)}), \dots, F_{2\phi(t^{(c)})+1}(t^{(c)})]$$
$$\text{and } B(t^{(c)}) = [B_1(t^{(c)}), B_2(t^{(c)}), \dots, B_{2\beta(t^{(c)})+1}(t^{(c)})],$$

where $\beta(t^{(c)})$ and $\phi(t^{(c)})$ are the number of backward and forward visits respectively.

So $F_{2i}(t^{(c)})$ and $F_{2i+1}(t^{(c)})$ are the forward entrance and exit times respectively for the ith forward visit. Also, $B_{2i+1}(t^{(c)})$ and $B_{2i}(t^{(c)})$ are the backward entrance and exit times respectively for the ith backward visit. $B_1(t^{(c)})$ and $F_1(t^{(c)})$ are the entrance and exit time of the visit upon which the circle is centred on.

So we have $B_{2\beta(t^{(c)})+1}(t^{(c)}) \leq \cdots \leq B_2(t^{(c)}) \leq B_1(t^{(c)}) \leq t^{(c)} \leq F_1(t^{(c)}) \leq F_2(t^{(c)}) \leq \cdots \leq F_{2\phi(t^{(c)})+1}(t^{(c)})$, which defines the animal's movements across the boundary of the circle centred at $t^{(c)}$. We define the total number of visits to be $\zeta(t^{(c)}) = 1 + \phi(t^{(c)}) + \beta(t^{(c)})$.

The point at which the animal first leaves a circle will be the centre of the next circle ($F_1(t_i^{(c)}) = t_{i+1}^{(c)}$). This means that the circle will be centred on the boundary of the previous circle.

We can now calculate the usage time to be

$$\psi(t^{(c)}) = F_1(t^{(c)}) - B_1(t^{(c)}) + \sum_{i=1}^{\phi(t^{(c)})} (F_{2i+1}(t^{(c)}) - F_{2i}(t^{(c)})) + \sum_{i=1}^{\beta(t^{(c)})} (B_{2i}(t^{(c)}) - B_{2i+1}(t^{(c)})).$$

S2.2. **Maximum percent drop.** Once the whole path is covered, we are left with a set of circles defined by their centres

$$\{(X_1^{(c)}, Y_1^{(c)}), (X_2^{(c)}, Y_2^{(c)}), \ldots, (X_{N_c}^{(c)}, Y_{N_c}^{(c)})\}$$

and their corresponding usage times $\{\psi_1, \psi_2, \ldots \psi_{N_c}\}$. We order these circles in descending order of usage time and remove any that overlap with a circle that has a higher usage time.

More precisely, after ordering them in descending order we have $\{\psi_{o_1}, \psi_{o_2}, \ldots \psi_{o_{N_c}}\}$ with $\psi_{o_i} \geq \psi_{o_{i+1}}$. Then we remove ψ_{o_i} if $d((X_{o_i}^{(c)}, Y_{o_i}^{(c)}), (X_{o_j}^{(c)}, Y_{o_j}^{(c)})) \leq 2R$ for any $j \in \{1, 2, \ldots i-1\}$. We are then reduced to a set of $\{\psi_{o'_1}, \psi_{o'_2}, \ldots, \psi_{o'_{N'}}\}$.

The maximum percent drop between consecutive usage times is defined as $MPD_R = \max\Big(1 - \frac{\psi_{o'_{i+1}}}{\psi_{o'_i}} \quad | \quad i \in \{1, 2, \ldots, N'-1\}\Big)$, where R is the radius used.

If $MPD_R = 1 - \frac{\psi_{o'_{j+1}}}{\psi_{o'_j}}$, the number of identified sites is j and they correspond to the circles with usage times, $\psi_{o'_1}, \psi_{o'_2}, \ldots, \psi_{o'_j}$. Also for the Ornstein-Uhlenbeck (OU) simulations we added the constraint that the identified sites cannot be all of the non-overlapping circles except for the first one. In that case the second highest percent drop was chosen, since the simulation started at this circle's centre, so would naturally have a lower usage time.

S2.2.1. *Brownian Motion Comparison.* We compare several Brownian motion simulations against some of our other paths with the aim of finding a minimum value for choosing the maximum percent drop threshold, T_{MPD}.

To be able to compare the Ornstein-Uhlenbeck (OU) and cattle paths with Brownian motion simulations, the step lengths for the Brownian motion simulations were drawn from an exponential distribution with the same mean as the path it is being compared with. Both paths are also the same length in time and have the same number of steps.

It can be seen in Figure S3 that the Brownian motion simulations have no clear drop whereas the OU ones do. It is this steep drop that someone using this algorithm should look for, both

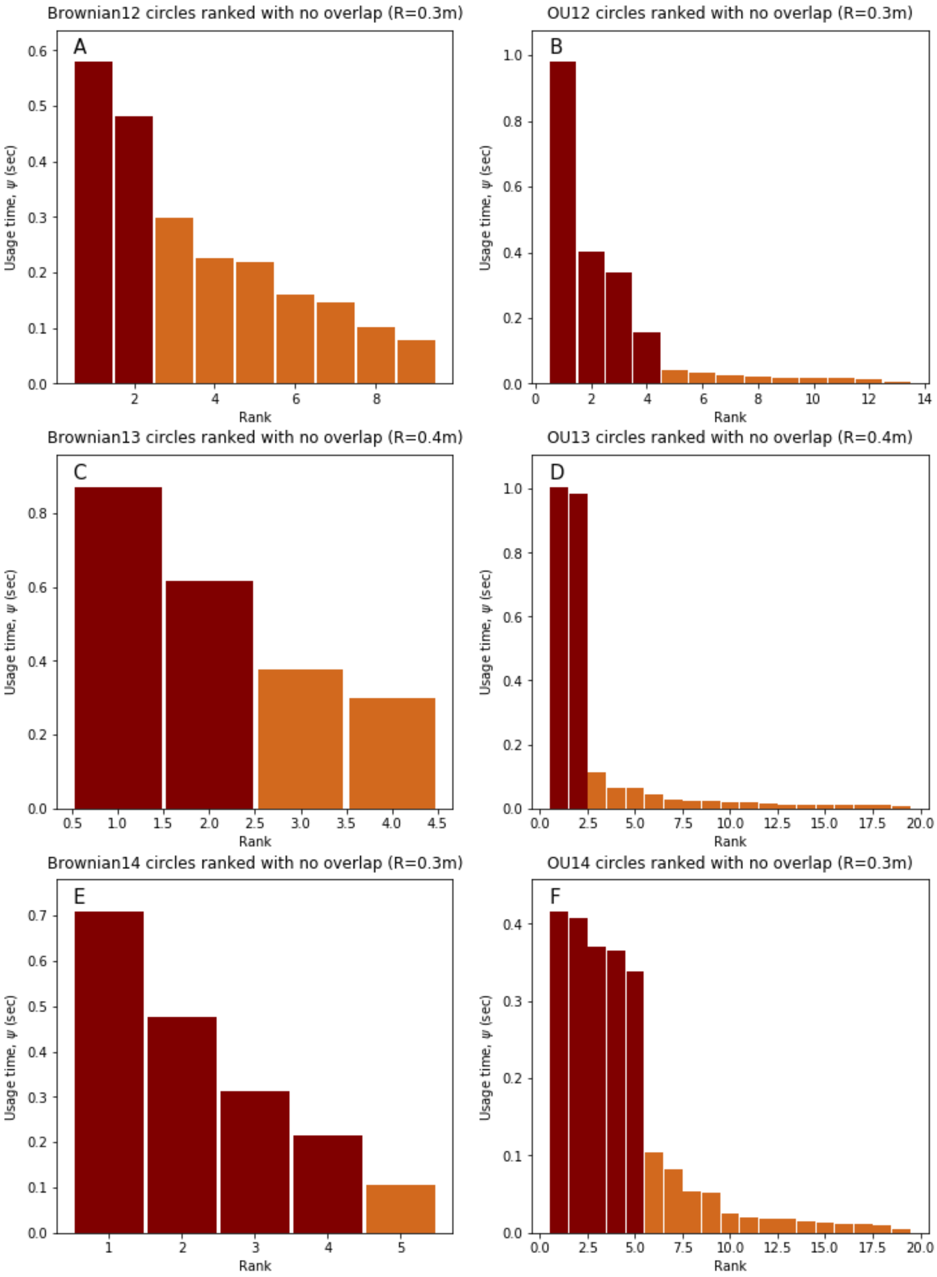

FIGURE S3. Bar plots of usage times comparing Brownian motion simulations (A, C and E) to OU ones (B, D and F).

in identifying the number of sites of interest and choosing which radius value to use. If the maximum percent drop (MPD) from the data was not notably higher than the one from the associated Brownian motion simulation then this may suggest that there are no sites of interest. A high MPD is indicative of a clearly distinct set of sites and we know a priori that a Brownian particle has no sites of interest, so we would expect the rank of residence times to be relatively smooth and the MPD to be low.

The same as for the OU simulations can be seen in Figure S4 for a particular cattle path. It should also be noted that for the Brownian motion simulations there are less circles, since the

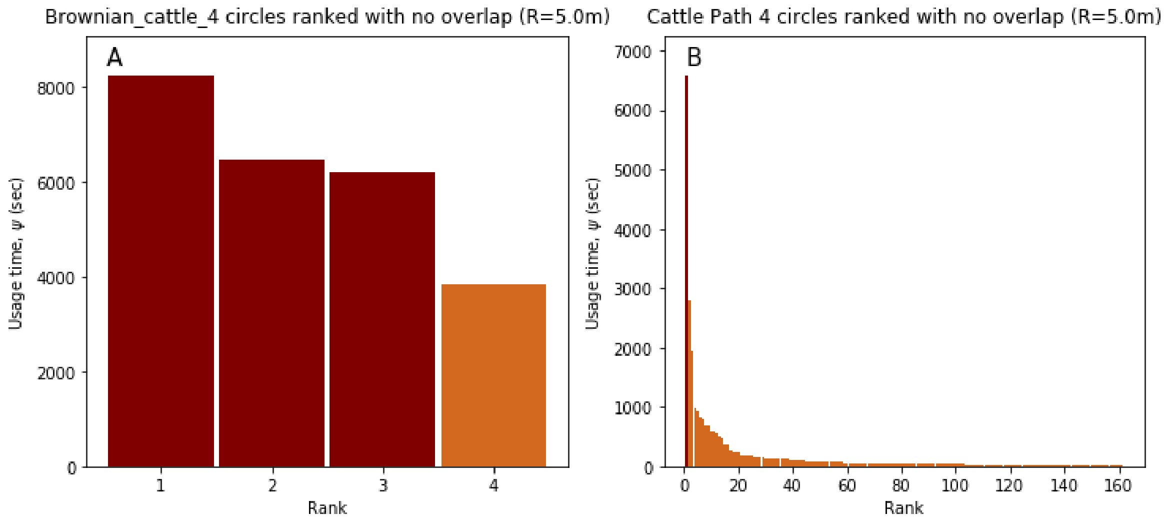

FIGURE S4. Bar plots of usage time for a Brownian motion simulation (A) compared to one from a cattle path (B).

paths for the Brownian motion simulations were more constrained and therefore it did not take as many circles to cover the whole path as it did for the OU simulations and the cattle paths (see Figures S3 & S4). This also restricted the number of possible values for the radius which could be used.

To give a lower bound for the threshold value for the cattle paths, we took the average of the maximum percent drops, excluding any radii values which resulted in a maximum percent drop of 100%. We found across all cattle paths this gave a lower bound of 47.5% and we used a threshold value of 50%.

S2.3. **Consistency Colour Assignment Explanation.** The first part of the algorithm will be repeated for different values of R, which are $\{R_{min}, R_{min}+r, R_{min}+2r, \ldots, R_{max}-r, R_{max}\}$. The second part of the algorithm involves choosing which of these values produces the best result. This is done using two criteria to choose the best R and then comparing the respective results.

The algorithm will search for the first local maximum percent drop, which is defined as $L(MPD_R) = \{MPD_{R^*} | MPD_{R^*} > MPD_{R^*-r}, MPD_{R^*} > MPD_{R^*+r} \text{ and } R^* \text{ is minimised}\}$.

We then incorporate a threshold criterion, defined as $MPD_R \geq T_{MPD}$. So

$$MPD_{R_1} = \{L(MPD_{R^*}) | MPD_{R^*} \geq T_{MPD}\}.$$

Alternatively, we can use an adaptive threshold value, meaning that $T_{MPD} = \min(MPD) + k(\max(MPD) - \min(MPD))$, where k is a constant and is referred to as the threshold ratio.

The stability criterion is defined as: R^* is stable if $n_{R^*-r} = n_{R^*} = n_{R^*+r}$, where n_R is the number of sites identified using the radius, R. So

$$MPD_{R_2} = \{L(MPD_{R^*}) | n_{R^*-r} = n_{R^*} = n_{R^*+r}\}.$$

Table S3 demonstrates how the confidence colour assignment works.

Red	Amber	Green
$n_{R_1} \neq n_{R_2}$	$n_{R_1} = n_{R_2}$ $R_1 \neq R_2$	$n_{R_1} = n_{R_2}$ $R_1 = R_2$

TABLE S3. The algorithm will assign a particular colour to the output value depending on the consistency between the two criteria, where n_R is the number of identified sites from using R. R_1 and R_2 are the identified radii from the threshold and stability criteria respectively.

S2.4. **Example.** Here we demonstrate our algorithm with a particular example. The actual number of sites for this simulation (OU64) is $\hat{n} = 3$.

In this example we have

- The results from taking the first local maximum are $R_0 = 0.3$ and $n_{R_0} = 133$.
- Adding a threshold value of 65% results in $R_1 = 0.8$ and $n_{R_1} = 3$.
- If the stability criterion is used, the results are $R_2 = 0.8$ and $n_{R_2} = 3$

Since the number of sites identified from using the threshold and stability criteria are the same, this path will be assigned either to the Amber or Green category. Then since the radii used to give these values are also the same, we can place this path in the Green category.

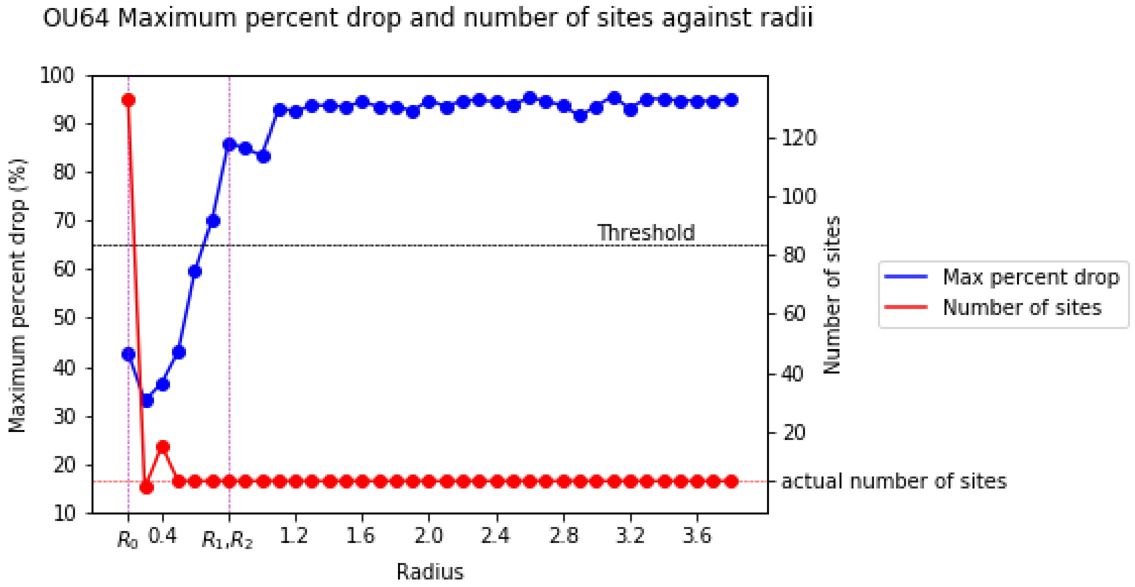

FIGURE S5. The first local maximum in the maximum percent drops over various radii when $R = R_0$. Adding the criterion that the maximum percent drop must be above a given threshold (65%) gives the radius as R_1. If the stability criterion is used, it gives the radius as R_2.

S3. Results

S3.1. **Ornstein Uhlenbeck details.** In Figure S6 we present four examples of OU paths assigned to each of the three colour categories and also indicate whether the correct number of sites was identified in the title of each subplot.

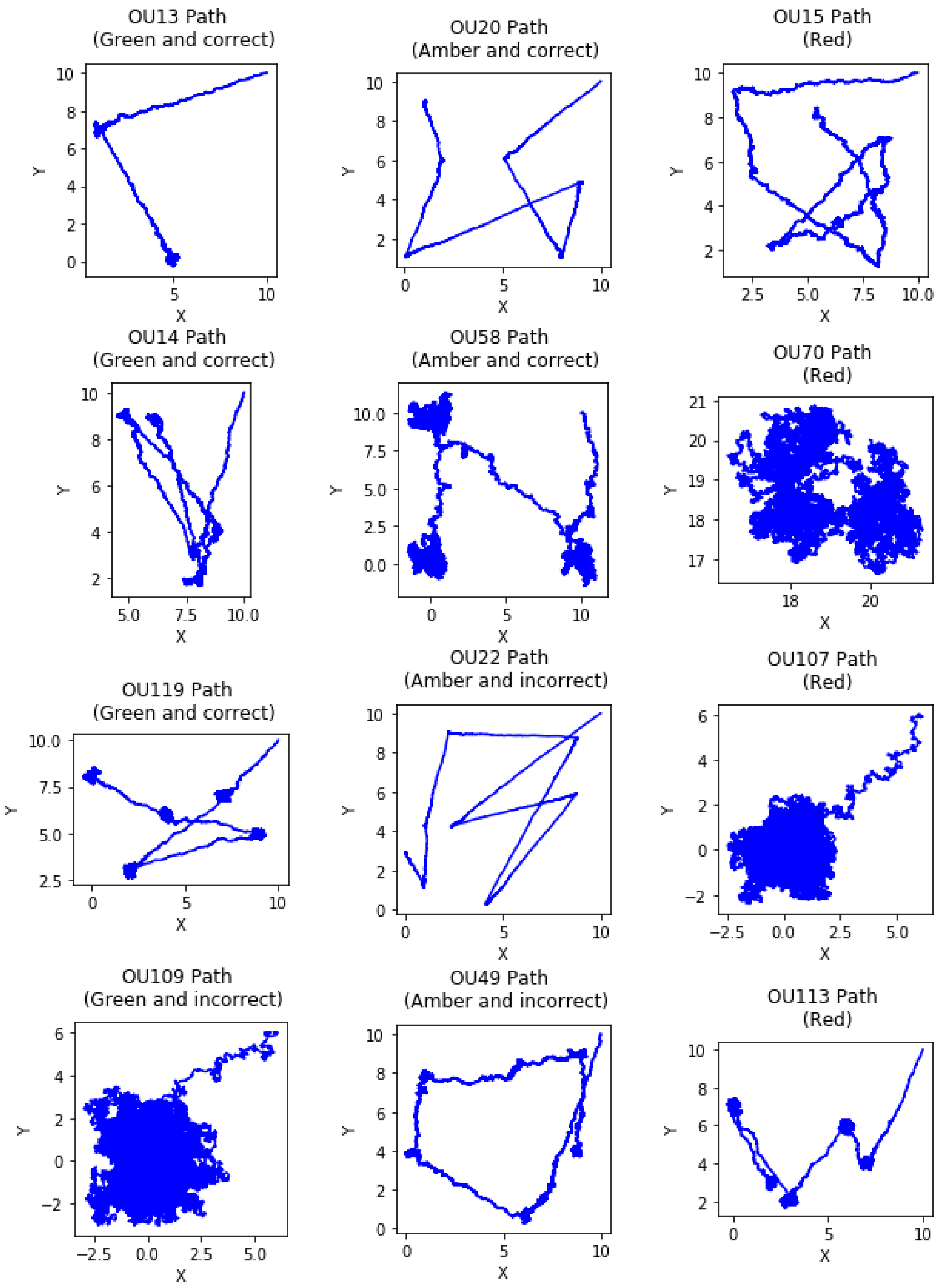

Figure S6. Different OU paths assigned to each of the 3 colour categories. The first column are assigned to the Green category, the second to the Amber category and the third to the Red category. Colours were assigned using a threshold value of 65%.

The colours were assigned using a threshold value of 65%, which was chosen so as to minimise the number of Green and incorrect paths. It also meant that for some simulations where the sites can be seen clearly by sight, such as OU107 and OU113 were in fact assigned as Red. This could suggest that a different threshold value would be better for these particular paths.

S3.2. **Cattle results.** Figure S7 is a screenshot of the sites we identified using a radius of 20m overlaid on Google maps. We can see that there is a wooded area between the milking station and sites to the South East, which explains why the cattle do not visit this area as often.

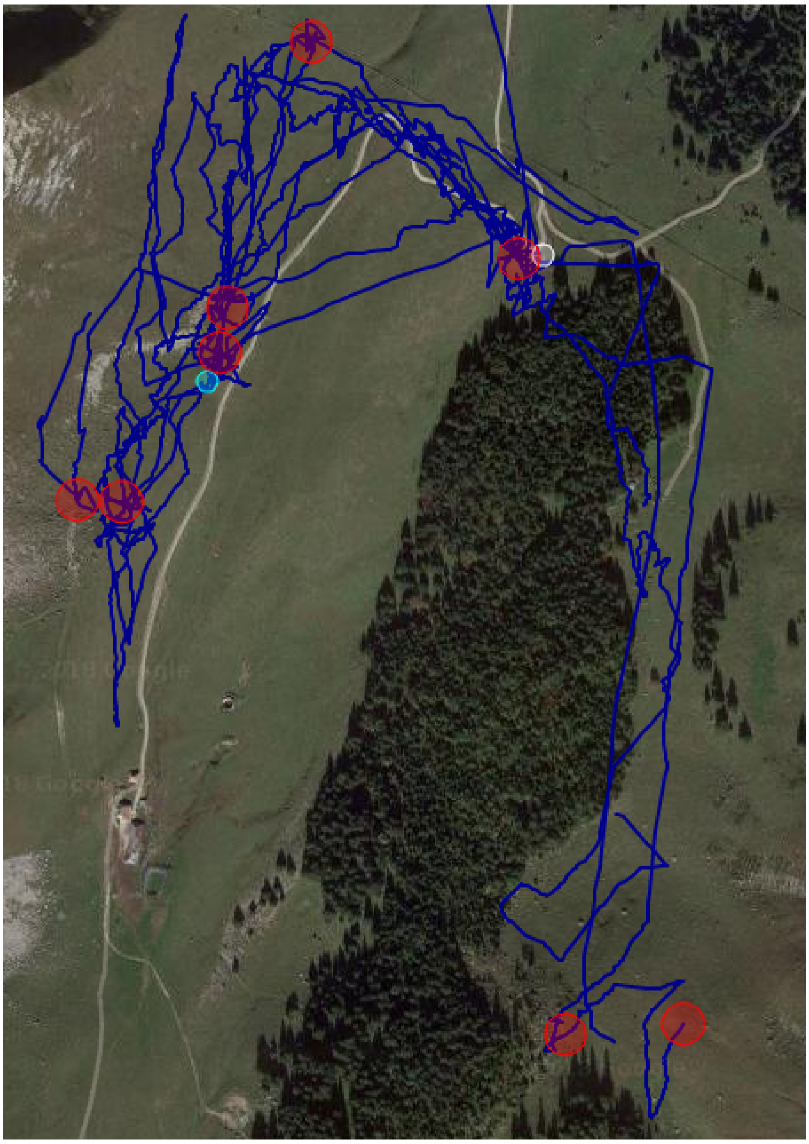

FIGURE S7. The seven cattle paths plotted on Google maps with the eight identified sites of interest in red with a radius of 20m, with the white dot and cyan dot representing the locations of the milking station and watering hole respectively.

In Figure S8 we present the original seven cattle paths along with the schematic representation for each day, when the radius used was 100m.

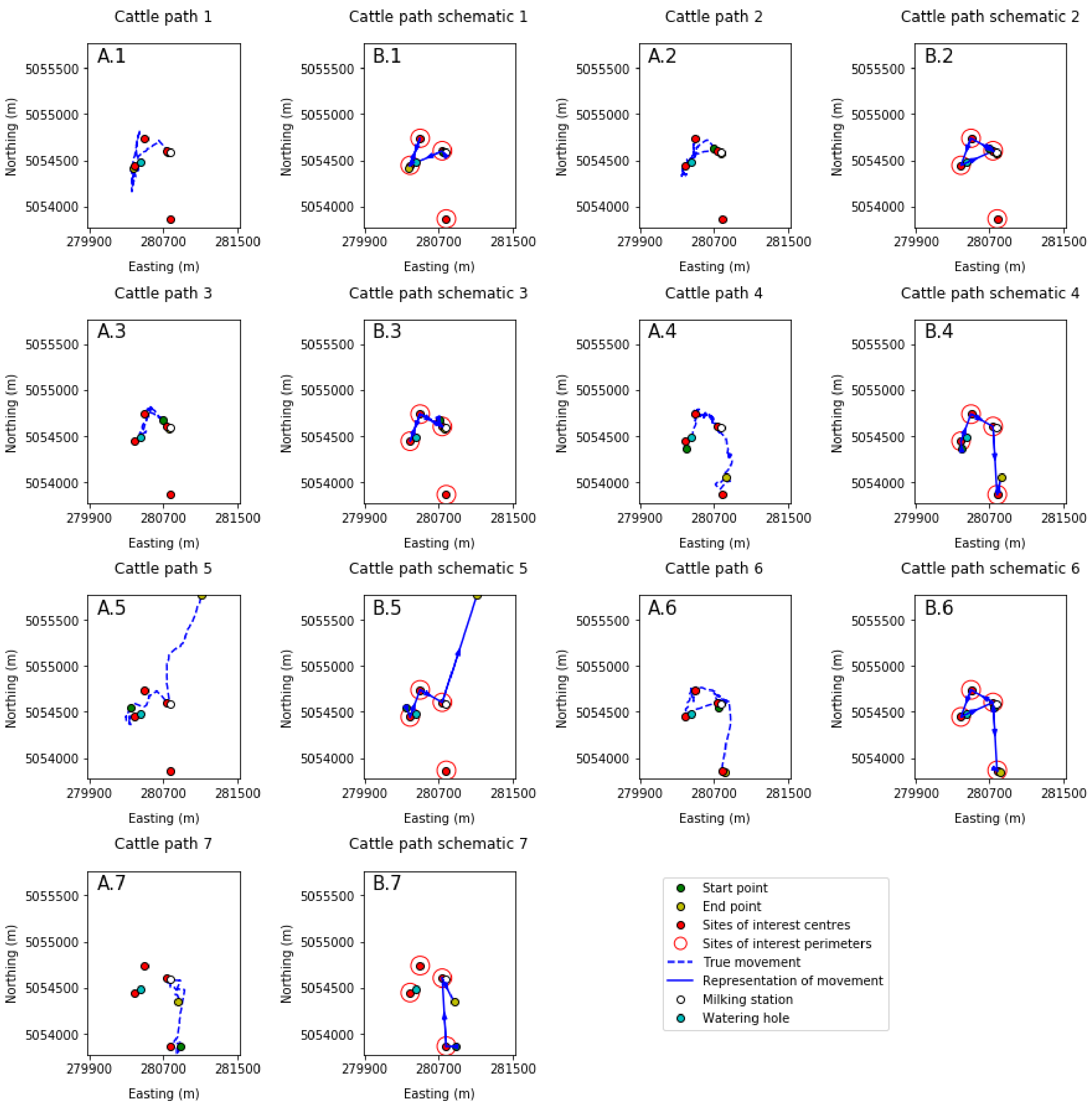

Figure S8. The paths for the Cattle for each of the seven paths (A.1-7) and the corresponding schematic plots (B.1-7) represent simplifications of these initial paths. The centres of sites of interest are defined by the red dots and their boundaries by the red hoops.

Radius (m)	Path 1	Path 2	Path 3	Path 4	Path 5	Path 6	Path 7
10	02:37.0	01:56.6	01:37.8	03:07.0	02:16.4	02:44.2	01:00.5
20	01:21.3	01:00.5	00:51.1	01:17.8	01:05.6	01:15.6	00:31.2
30	00:49.6	00:37.8	00:29.9	00:41.3	00:40.6	00:39.4	00:20.8
40	00:27.5	00:25.8	00:23.6	00:22.8	00:25.9	00:23.5	00:17.4
50	00:20.5	00:17.7	00:14.1	00:18.1	00:19.0	00:17.6	00:12.5
60	00:15.4	00:15.0	00:10.5	00:12.4	00:13.2	00:13.7	00:09.2
70	00:12.4	00:10.8	00:09.4	00:10.4	00:11.3	00:10.9	00:09.2
80	00:10.4	00:08.4	00:07.3	00:06.8	00:09.0	00:08.6	00:07.5
90	00:08.1	00:06.7	00:07.7	00:06.3	00:08.5	00:06.9	00:07.0
100	00:06.7	00:05.7	00:06.7	00:05.6	00:07.8	00:05.8	00:05.0
Average	00:38.9	00:30.5	00:25.8	00:38.9	00:33.7	00:36.6	00:18.0

Table S4. The runtimes (min:sec) from applying the algorithm to cattle data for seven paths and using $s = 10$, which is the length of interval that the algorithm looks over.

Radius (m)	Path 1	Path 2	Path 3	Path 4	Path 5	Path 6	Path 7
10	14:22.5	20:54.4	16:24.3	29:45.8	21:28.4	26:03.4	18:42.4
20	06:47.2	09:43.0	07:48.1	13:04.8	10:26.6	12:14.4	08:38.2
30	04:25.4	05:49.5	04:55.1	06:15.9	06:44.9	06:45.1	05:36.9
40	03:05.4	03:58.8	03:35.0	03:19.1	04:26.7	04:07.7	03:54.9
50	02:28.1	02:34.1	02:18.2	02:30.5	03:06.3	02:34.1	02:49.4
60	02:00.8	01:58.3	01:48.1	01:43.4	02:17.4	01:58.0	02:01.4
70	01:39.8	01:41.3	01:34.6	01:33.3	01:42.8	01:34.7	01:39.4
80	01:24.9	01:22.5	01:10.7	01:05.5	01:18.9	01:17.7	01:31.4
90	01:13.9	00:53.2	01:10.2	00:58.1	01:09.6	01:08.8	01:18.1
100	00:58.7	00:43.5	01:06.0	00:51.6	01:05.8	00:57.4	01:04.6
Average	03:50.7	04:57.9	04:11.0	06:06.8	05:22.7	05:52.1	04:43.7
Ratio between averages for s=1 and 10	5.93	9.77	9.73	9.43	9.57	9.62	15.76

Table S5. The runtimes (min:sec) from applying the algorithm to cattle data for seven paths and using $s = 1$, which is the length of interval that the algorithm looks over.

OU number	Number of points of attraction	Long term standard deviation	Average distance between points of attraction
11	2	17.32050808	4.123105626
12	1	25.29822128	0
13	2	17.5	8.062257748
14	5	15.68929081	4.083655616
15	9	21.38089935	5.425134867
16	3	14.43375673	7.038119745
17	7	18.89822365	4.49114932
18	9	8.528028654	3.399783517
19	8	15.11857892	3.85324933
20	6	7.071067812	5.382584744
21	2	2.357022604	3.605551276
22	8	4.714045208	5.556525929
23	8	6.123724357	5.076562436
24	1	18.89822365	0
25	7	8.660254038	4.912798158
26	5	10	5.725744631
27	2	22.36067978	8.062257748
28	4	15.68929081	7.095093289
29	5	28.86751346	4.976695174
30	9	6.123724357	4.729544651
31	6	17.32050808	4.064343555
32	3	2.236067978	3.954624491
33	3	14.14213562	4.717943447
34	4	18.85618083	3.903077327
35	3	2.236067978	8.398751316
36	5	2.886751346	3.127913027
37	7	7.844645406	5.889149455
38	7	9.805806757	4.612522825

TABLE S6. Table of how the OU simulations (11-38) vary, including the number of points of attraction, the long term standard deviation about these points and the average distance between points of attraction.

OU number	Number of points of attraction	Long term standard deviation	Average distance between points of attraction
39	6	8.944271910	4.671099195
40	4	25.29822128	4.059632853
41	5	8.528028654	2.601533972
42	1	26.72612419	0
43	9	25	6.194382572
44	2	5.773502692	4.472135955
45	7	26.72612419	6.407601193
46	7	6.123724357	4.562102360
47	4	14.28869017	7.570496844
48	5	17.00840129	3.370118293
49	7	22.36067978	5.864138580
50	1	16.03567451	0
51	3	18.25741858	11.38071187
52	3	25.56038602	11.38071187
53	3	32.86335345	11.38071187
54	3	40.16632088	11.38071187
55	3	47.46928832	11.38071187
56	3	54.77225575	11.38071187
57	3	62.07522318	11.38071187
58	3	69.37819062	11.38071187
59	3	76.68115805	11.38071187
60	3	83.98412548	11.38071187
61	3	54.77225575	22.76142375
62	3	54.77225575	20.48528137
63	3	54.77225575	18.20913900
64	3	54.77225575	15.93299662
65	3	54.77225575	13.65685425
66	3	54.77225575	11.38071187

Table S7. Table of how the OU simulations (39-66) vary, including the number of points of attraction, the long term standard deviation about these points and the average distance between points of attraction.

OU number	Number of points of attraction	Long term standard deviation	Average distance between points of attraction
67	3	54.77225575	9.104569500
68	3	54.77225575	6.828427125
69	3	54.77225575	4.552284750
70	3	54.77225575	2.276142375
71	3	17.67766953	1.885618083
72	3	17.67766953	3.771236166
73	3	17.67766953	3.771236166
74	3	17.67766953	3.771236166
75	3	17.67766953	3.771236166
76	3	17.67766953	3.771236166
77	3	17.67766953	3.771236166
78	3	17.67766953	3.771236166
79	3	17.67766953	3.771236166
80	3	17.67766953	3.771236166
81	3	27.38612788	5.841619253
82	3	27.38612788	6.222238329
83	3	27.38612788	4.066930952
84	3	27.38612788	4.520207766
85	3	27.38612788	5.997850602
86	3	27.38612788	6.632054515
87	3	27.38612788	4.976702199
88	3	27.38612788	4.867042053
89	3	27.38612788	5.575760139
90	3	27.38612788	5.079001964
91	3	27.38612788	22.76142375
92	3	27.38612788	20.48528137
93	3	27.38612788	18.20913900

TABLE S8. Table of how the OU simulations (67-93) vary, including the number of points of attraction, the long term standard deviation about these points and the average distance between points of attraction.

OU number	Number of points of attraction	Long term standard deviation	Average distance between points of attraction
94	3	27.38612788	15.93299662
95	3	27.38612788	13.65685425
96	3	27.38612788	11.38071187
97	3	27.38612788	9.1045695
98	3	27.38612788	6.828427125
99	3	27.38612788	4.55228475
100	3	27.38612788	2.276142375
101	1	0.1	0
102	1	0.3	0
103	1	0.5	0
104	1	0.7	0
105	1	0.9	0
106	1	1.1	0
107	1	1.3	0
108	1	1.5	0
109	1	1.7	0
110	1	1.9	0
111	5	13.69306394	6.178002262
112	5	13.69306394	4.563538614
113	5	13.69306394	3.473467008
114	5	13.69306394	5.326197512
115	5	13.69306394	3.219039168
116	5	13.69306394	5.891979101
117	5	13.69306394	6.526701595
118	5	13.69306394	4.674765813
119	5	13.69306394	6.039779245
120	5	13.69306394	3.711774791

Table S9. Table of how the OU simulations (94-120) vary, including the number of points of attraction, the long term standard deviation about these points and the average distance between points of attraction.

OU number	Number of points of attraction	Identified sites from Threshold (0.7)	Identified sites from Stable	Radius from Threshold	Radius from Stable	Colour category
11	2	2	2	0.4	0.4	Green
12	1	1	1	0.5	0.5	Green
13	2	2	2	0.4	0.4	Green
14	5	5	5	0.3	0.3	Green
15	9	7	2	1.1	0.3	Red
16	3	3	3	0.5	0.5	Green
17	7	7	7	0.4	0.4	Green
18	9	5	1	0.9	2	Red
19	8	7	1	0.7	1.8	Red
20	6	6	6	0.6	0.3	Amber
21	2	2	1	0.2	1.3	Red
22	8	1	1	3.5	1.6	Amber
23	8	8	1	1	2	Red
24	1	1	1	0.9	0.9	Green
25	7	5	3	1.2	1.5	Red
26	5	5	5	0.4	0.4	Green
27	2	2	2	0.3	0.3	Green
28	4	4	4	0.5	0.5	Green
29	5	3	2	1.1	1.7	Red
30	9	6	2	0.9	1.9	Red
31	6	4	1	1.2	1.9	Red
32	3	3	3	0.4	0.4	Green
33	3	3	3	0.2	0.4	Amber
34	4	6	2	0.4	1	Red
35	3	3	3	0.4	0.4	Green
36	5	5	3	0.5	1.4	Red
37	7	7	6	0.4	1.1	Red
38	7	5	1	0.6	2.3	Red

TABLE S10. The results from applying the algorithm to OU simulations 11 - 38.

OU number	Number of points of attraction	Identified sites from Threshold (0.7)	Identified sites from Stable	Radius from Threshold	Radius from Stable	Colour category
39	6	5	6	0.8	0.4	Red
40	4	5	4	0.9	1.2	Red
41	5	5	5	0.3	0.3	Green
42	1	1	1	0.8	0.8	Green
43	9	8	1	1	2.7	Red
44	2	2	2	0.3	0.3	Green
45	7	8	3	0.9	1.7	Red
46	7	15	1	0.5	1.2	Red
47	4	4	4	0.5	0.5	Green
48	5	5	1	0.4	2	Red
49	7	6	6	1.1	0.8	Amber
50	1	1	1	0.6	0.6	Green
51	3	3	3	0.5	0.5	Green
52	3	3	3	0.5	0.5	Green
53	3	3	3	0.7	0.7	Green
54	3	3	3	0.8	0.8	Green
55	3	3	3	0.8	0.8	Green
56	3	3	3	0.6	0.6	Green
57	3	3	3	0.8	0.8	Green
58	3	3	3	1	0.6	Amber
59	3	3	3	1.2	1.2	Green
60	3	3	3	0.7	0.7	Green
61	3	3	3	1	1	Green
62	3	3	3	1.1	1.1	Green
63	3	3	3	0.9	0.9	Green
64	3	3	3	0.8	0.8	Green
65	3	3	3	0.9	0.9	Green
66	3	3	3	1	1	Green

TABLE S11. The results from applying the algorithm to OU simulations 39 - 66.

OU number	Number of points of attraction	Identified sites from Threshold (0.7)	Identified sites from Stable	Radius from Threshold	Radius from Stable	Colour category
67	3	3	3	0.7	0.7	Green
68	3	3	3	0.8	0.8	Green
69	3	3	3	0.7	0.7	Green
70	3	22	3	0.3	0.8	Red
71	1	1	1	0.8	0.8	Green
72	2	2	2	0.7	0.7	Green
73	3	3	3	0.3	0.3	Green
74	4	4	4	0.3	0.3	Green
75	5	5	5	0.3	0.3	Green
76	6	6	6	0.3	0.3	Green
77	7	7	7	0.3	0.3	Green
78	8	8	8	0.7	0.7	Green
79	9	9	9	0.7	0.7	Green
80	10	10	10	0.4	0.4	Green
81	3	3	3	0.6	0.6	Green
82	3	3	3	0.5	0.5	Green
83	3	3	3	0.5	0.5	Green
84	3	3	3	0.7	0.7	Green
85	3	3	3	0.6	0.6	Green
86	3	3	3	0.7	0.7	Green
87	3	3	3	0.7	0.7	Green
88	3	3	3	0.7	0.7	Green
89	3	3	3	0.6	0.6	Green
90	3	3	3	0.6	0.6	Green
91	3	3	3	0.3	0.3	Green
92	3	3	3	0.4	0.4	Green
93	3	3	3	0.4	0.4	Green

TABLE S12. The results from applying the algorithm to OU simulations 67 - 93.

OU number	Number of points of attraction	Identified sites from Threshold (0.7)	Identified sites from Stable	Radius from Threshold	Radius from Stable	Colour category
94	3	3	3	0.3	0.3	Green
95	3	3	3	0.5	0.5	Green
96	3	3	3	0.5	0.5	Green
97	3	3	3	0.4	0.4	Green
98	3	3	3	0.5	0.5	Green
99	3	3	3	0.3	0.3	Green
100	3	3	3	0.3	0.3	Green
101	1	1	1	0.4	0.4	Green
102	1	1	1	0.4	0.4	Green
103	1	5	1	0.3	0.6	Red
104	1	7	1	0.4	0.9	Red
105	1	6	1	0.4	1	Red
106	1	6	1	0.6	1.2	Red
107	1	7	1	0.7	1.5	Red
108	1	12	1	0.7	1.3	Red
109	1	6	6	1	1	Green
110	1	8	1	0.9	2	Red
111	5	5	5	0.4	0.4	Green
112	5	5	5	0.4	0.4	Green
113	5	5	3	0.6	1.3	Red
114	5	5	5	0.5	0.5	Green
115	5	5	5	0.3	0.3	Green
116	5	5	5	0.5	0.5	Green
117	5	5	5	0.5	0.5	Green
118	5	5	5	0.7	0.7	Green
119	5	5	5	0.4	0.4	Green
120	5	5	5	0.5	0.5	Green

TABLE S13. The results from applying the algorithm to OU simulations 94 -120.

REFERENCES

Benhamou, S. and L. Riotte-Lambert. 2012. Beyond the Utilization Distribution: Identifying home range areas that are intensively exploited or repeatedly visited. Ecological Modelling, **227**:112–116.

Cattle path	Identified sites from Threshold (0.5)	Identified sites from Stable	Radius from Threshold	Radius from Stable	Colour category
1	3	3	11.4	11.4	Green
2	2	2	14	16	Amber
3	2	2	12	12	Green
4	1	1	10	11	Amber
5	24	7	12	36	Red
6	2	2	10	13	Amber
7	2	7	10.5	20	Red

Table S14. The results from applying the algorithm to cattle data for seven paths.
